# The Spectrum of Postoperative Complications and Outcomes After Open Adrenalectomy: An Experience From a Developing Country

**DOI:** 10.7759/cureus.31357

**Published:** 2022-11-11

**Authors:** Abdullah Bin Zubair, Muhammad Haad Arif, Mustafa Tauseef Razzaq, Maham Zaman, Zaki Haider, Ibtesam-e Fajar, Samra Saleem, Adil Khalil, Muzammil Sabir, Mehwish Kaneez

**Affiliations:** 1 Research, Mayo Clinic, Rochester, USA; 2 Surgery, Shalamar Medical and Dental College, Lahore, PAK; 3 Surgery, Rashid Latif Medical College, Lahore, PAK; 4 Surgery, Bahawal Victoria Hospital, Bahawalpur, PAK; 5 Surgery, Al Nafees Medical College and Hospital, Islamabad, PAK; 6 Surgery, Rawalpindi Medical University, Rawalpindi, PAK

**Keywords:** postoperative complications, postoperative outcomes, adrenal pheochromocytoma, adrenal gland neoplasms, open adrenalectomy

## Abstract

Background

Open adrenalectomy is an invasive surgical procedure that is commonly performed for adrenal gland neoplasms in developing countries. Due to its complexity, the patients are predisposed to a number of complications and dismal outcomes. The objective of our study is to assess different characteristics of patients undergoing open adrenalectomy, including their histology, postoperative complications, and outcomes.

Methods

This retrospective cross-sectional study included 107 patients undergoing open adrenalectomy for primary adrenal gland neoplasms. Patients with bilateral involvement, metastatic disease, or unresectable tumors were excluded. Patients were evaluated for different features that included demographic data, tumor properties, postoperative outcomes, and complications.

Results

Out of 107 patients, 45 (42.1%) were females. The mean age of the patients was 47.53 ± 8.45 years. Abdominal pain and severe headaches were the most common presenting complaints. A total of 96 (89.7%) tumors were benign, while 11 (10.3%) were malignant. Upon the histopathological examination of the resected specimen, adrenal adenoma was present in 49 (45.8%) cases, while adrenal pheochromocytoma was present in 41 (38.3%) cases. A total of 51 patients developed different postoperative complications including surgical site infections (22.4%), atelectasis (11.2%), deep venous thrombosis (7.5%), and retroperitoneal hematoma (5.6%). In-hospital mortality occurred in three (2.8%) patients.

Conclusion

Surgical site infections, atelectasis, deep venous thrombosis, and retroperitoneal hematoma were frequent postoperative complications after open adrenalectomy. These complications increase morbidity and mortality, especially in developing countries. Improved surgical techniques, intraoperative hemostasis, and multidisciplinary approach can yield favorable postoperative outcomes.

## Introduction

Tumors of the adrenal gland are a heterogenous group of neoplastic entities that possess a highly sporadic nature [1]. These tumors can arise from the adrenal cortex or the medulla and can cause endocrine dysfunction. The prevalence of adrenal gland tumors ranges between 1% and 4% of all endocrine gland tumors [2]. These tumors can be benign adenomas or life-threatening malignant carcinomas [3]. Nearly 80% of adrenal gland tumors are benign, while around 8% are reported to be malignant [1,3].

Adrenal gland tumors are divided into adrenocortical tumors and adrenomedullary tumors [4]. The most common primary adrenal tumors are adrenocortical adenomas; however, their malignant counterpart adrenocortical carcinomas are relatively rare with a poor prognosis [5]. The incidence of adrenocortical carcinoma lies between 0.5 and two per million people worldwide [2,5]. Adrenal tumors can be nonfunctional or functional. Functional adrenal gland tumors may produce aldosterone, cortisol, catecholamines, or sex hormones [6]. Thus, patients with the functional adrenal mass present with specific endocrinological signs and symptoms. These include hypertension, headache in pheochromocytoma, electrolyte disturbances in Conn’s syndrome (hypernatremia and hypokalemia), and features of Cushing’s disease in adrenocortical tumors [3]. Moreover, pain, fatigue, weight loss, and abdominal fullness are some of the nonspecific symptoms. Nearly 10% of adrenal adenomas are asymptomatic and are diagnosed incidentally on imaging.

Before the emergence and advancement of radiographic techniques (CT and MRI scans), adrenal tumors were considered a rare entity. Therefore, it was difficult to diagnose them unless they were functional or reached a size that compressed adjacent organs [7]. Currently, meticulous analytical skills and detailed radiographic techniques have improved the early detection rates of adrenal gland tumors [8]. This allows the surgeons to accurately locate and characterize the lesion. Moreover, it helps in the accurate delineation and staging of the disease, which are necessary for the decision of optimal surgical treatment [9].

Surgical resection is considered the gold standard treatment for benign and most early-stage malignant tumors. For several decades, open adrenalectomy was the treatment of choice for adrenal tumors [8,9]. However, minimally invasive laparoscopic adrenalectomy has instantly gained popularity due to fewer complications [10]. The literature reports that laparoscopic adrenalectomy is linked with decreased blood loss, shorter hospital stays, and fewer intraoperative and postoperative complications. In contrast, open adrenalectomy is associated with a large incision, delayed healing, lengthy recovery, and relatively high intraoperative and postoperative complications [6,10,11]. Henceforth, laparoscopic adrenalectomy is considered the better approach for adrenal tumors. Unfortunately, the cost of the procedure, limited resources, expensive equipment, and the lack of trained surgical faculty have made the use of laparoscopic adrenalectomy limited in developing countries [8,9]. As a result, open adrenalectomy is more commonly practiced in public sector hospitals in developing countries such as Pakistan. As a result, patients experience a number of postoperative complications that significantly affect the postoperative outcomes and survival rates [12].

It is important to precisely evaluate the prevalence of these postoperative complications so that appropriate measures can be taken to avoid them. Moreover, the low research output in developing countries along with the lack of resources necessitates the requirement for our study so that the prevalence of these postoperative complications can be ascertained and necessary steps are taken to reduce them. Therefore, this study targets to evaluate the postoperative outcomes and complications following open adrenalectomy.

## Materials and methods

This retrospective cross-sectional study was conducted at the Department of General Surgery, District Headquarter Hospital, Rawalpindi, Pakistan. The study includes all the patients who underwent unilateral open adrenalectomy from March 2015 to March 2021 (six years). All the patients who presented with hyperfunctioning adrenal mass or incidentaloma on imaging were included in the study. Patients with incomplete reported data and those operated via laparoscopic approach were excluded. Additionally, patients with severe multi-visceral involvement and the presence of metastatic disease were also excluded. Finally, a total of 107 patients were part of the final data analysis. The institutional review board of Rawalpindi Medical University granted ethical approval with the approval number IRB-2022-SUR-129.

Consultant surgeons with more than five years of experience and who had completed at least 30 open adrenalectomies operated on all the patients. Data collection was completed using clinical notes and computer records. Patients were evaluated based on their demographic data, American Society of Anesthesiologists (ASA) classification, primary diagnosis, length of hospital stay, tumor site, grading, and histopathological subtype. The postoperative outcomes and the prevalence of various postoperative complications were also ascertained. The data were entered and analyzed using Statistical Package for Social Sciences (SPSS) (IBM SPSS Statistics, Armonk, NY) version 25. Categorical variables were expressed as frequency and percentages, while numerical variables were assessed as mean and standard deviation.

## Results

In the present study analyzing 107 patients, the mean age was 47.53 ± 8.45 years with a range of 36-64 years. The majority of the patients had multiple presenting symptoms. The demographic details of the study participants are elucidated in Table 1.

**Table 1:** Demographic details of study participants.

Parameters		Frequency	Percentages
Gender	Male	62	57.9%
	Female	45	42.1%
Age groups	30-40 years	27	25.2%
	41-50 years	49	45.8%
	51-60 years	22	20.6%
	60-70 years	9	8.4%
Presenting symptoms	Fatigue	34	31.8%
	Vomiting	29	27.1%
	Abdominal pain	67	62.6%
	Unexplained weight loss	14	14.9%
	Palpable abdominal mass	22	20.6%
	Severe headaches	36	33.65
	Weight gain	12	11.2%
Comorbidities	Diabetes mellitus	16	14.9%
	Hypertension	14	13.1%
	Ischemic heart disease	5	4.7%
	Asthma	3	2.8%
	No comorbidities	69	64.5%

Most of the patients had an ASA score of II. A total of 74 (69.2%) of the operated cases had an endocrine activity of which 41 (38.3%) were catecholamine-secreting tumors, 22 (20.6%) were aldosterone-secreting tumors, and 11 (10.3%) were glucocorticoid-secreting tumors. These patient characteristics are defined in Table 2.

**Table 2:** A tabulation of operative characteristics of study participants. ASA: American Society of Anesthesiologists

Parameter		Frequency	Percentages
ASA score	II	89	83.2%
	III	12	11.2%
	IV	6	5.6%
History of previous abdominal surgery	Yes	6	5.6%
	No	101	94.4%
Resection side	Right	58	54.2%
	Left	49	45.8%
Nature of tumor	Benign	96	89.7%
	Malignant	11	10.3%
Endocrine activity	Catecholamines	41	38.3%
	Aldosterone	22	20.6%
	Glucocorticoid	11	10.3%
	Absent	33	30.8%
Surgical approach	Transabdominal	50	46.7%
	Retroperitoneal	57	53.3%

Pheochromocytoma was found to be the most common histopathological subtype found among 41 (38.3%) of the operative cases. A total of 11 (10.3%) patients had malignant adrenocortical carcinoma. Further highlights of histopathological subtypes of the resected tumors are shown in Table 3.

**Table 3:** A tabulation of histopathological details of the resected tumors.

Histopathological subtype	Frequency	Percentages
Pheochromocytoma	41	38.3%
Adrenocortical adenoma	49	45.8%
Adrenocortical carcinoma	11	10.3%
Nodular hyperplasia	3	2.8%
Ganglioneuroma	2	1.9%
Corticomedullary mix tumor	1	0.9%

A total of 54 (50.5%) of the patients had an unremarkable postoperative course with no complications and were discharged within one week after surgery. Surgical site infection was the most common complication observed in a total of 24 (22.4%) of the patients, while retroperitoneal hematoma was observed in a total of six (5.6%) cases. A spectrum of postoperative complications is delineated in Table 4.

**Table 4:** Postoperative complications among the included patients.

Postoperative complications	Frequency	Percentages
Surgical site infection	24	22.4%
Pneumonia/atelectasis	12	11.2%
Deep venous thrombosis	8	7.5%
Retroperitoneal hematoma	6	5.6%
Pulmonary embolism	2	1.9%
Sepsis	1	0.9%
Total	53	49.5%

The mean length of hospital stay was 5.47 ± 3.21 days. Blood transfusion was required in 38 (35.5%) patients because of low hemoglobin levels. In-hospital mortality was observed in three (2.8%) cases. Moreover, one patient had to be re-explored due to the formation of massive retroperitoneal hematoma and excessive bleeding but unfortunately couldn’t survive. These postoperative outcomes are shown in Table 5.

**Table 5:** Postoperative outcomes of the study cohort.

Postoperative outcomes	Frequency	Percentages
Blood transfusion	38	35.5%
In-hospital mortality	3	2.8%
Mechanical ventilatory support	3	2.8%
Need for re-exploration	1	0.9%

## Discussion

In developing countries with a lack of the latest equipment and trained surgeons, the rate of invasive surgeries such as open adrenalectomy is significantly high. It involves complete resection of the adrenal glands via a transabdominal or retroperitoneal approach [13]. Due to its invasive nature, it has several procedural complications that negatively impact surgical outcomes. Nonetheless, the procedure is still curative for various adrenal gland tumors [11].

Our study showed that 45.8% of the patients with adrenal mass were aged between 41 and 50 years, while the mean age of all study participants was 47.52 years. A similar study from Saudi Arabia on 55 patients showed similar results where the mean age of patients was 49.91 years [9]. Other studies also report similar findings where more than 35% of the patients were aged between 41 and 50 years [4,14]. Abdominal pain and severe headaches were the most frequent presenting complaints of the patients. Frequent severe headaches are due to high blood pressure secondary to the catecholamine surge in pheochromocytoma [2,4]. Pertinently, abdominal pain is due to a developing adrenal mass [4]. The majority of the patients in our study had an ASA score of II; i.e., most patients undergoing surgery had a mild systemic illness but had no functional limitations. This is similar to another study from Africa that reported the mean ASA classification to be 2.6 ± 0.57 [8].

The prevalence of malignant adrenal tumors is reported to be rare in the literature [5]. Our study shows a similar pattern where 89.7% of the patients had benign adrenal tumors, while 10.3% underwent a malignant transformation. Studies have demonstrated that early resection is an important step in the management of surgically resectable primary malignant adrenal tumors [1,5]. Timely resection of these tumors can significantly improve overall survival and prevent morbidity. Clinical and histopathological analyses of the resected tumors revealed that pheochromocytoma was the most common hormone-secreting tumor, followed by aldosterone-secreting tumors and steroid-secreting adrenal tumors. Other studies from developing countries yield similar results with an increased prevalence of catecholamine-secreting tumors [7-9,15]. Pheochromocytomas typically present with severe headaches and hypertension secondary to the catecholamine surge [4]. A systematic review shows that the overall recurrence rate of these tumors after resection is only 3%, and these tumors show good survival rates after resection [16].

Patients in our study showed a high postoperative complication rate of 49.5%. Surgical site infection was the most frequent postoperative complication observed among our patients. Surgical site infections are an extremely common yet preventable cause of morbidity and mortality especially in developing countries [17]. These infections disseminate and lead to severe pneumonia or sepsis, which further contributes to morbidity and increases the length of hospital stay [18]. Moreover, postoperative pneumonia and sepsis could also be prevented with appropriate surgical antimicrobial prophylaxis and strict infection control measures. Optimal postoperative surgical care is of paramount importance in preventing such postoperative complications; however, in developing countries, the lack of resources and poor infection control measures lead to abysmal outcomes and a high complication rate [8,12]. Retroperitoneal hematomas were observed in a total of six patients. Meticulous intraoperative hemostasis management and accurate operative technique are the greatest challenges during open adrenalectomy [19]. Poor intraoperative hemostasis leads to hematoma formation that may ultimately translate into hypovolemic shock or re-exploration [10,14]. Improvement in surgical techniques and limiting blood loss can significantly reduce this complication.

Even with the difficulty and intricacy of the procedure, these postoperative complications can be reduced with strict infection control protocols. Moreover, an extensive evaluation of all patients before the surgery can further help to improve postoperative outcomes. Appropriate preoperative optimization of patients before the procedure and the administration of prophylactic antibiotics according to the latest guidelines can significantly reduce the incidence of surgical site infections, postoperative pneumonia, and sepsis [17]. Moreover, the rational administration of antibiotics along with adherence to antibiotic stewardship programs can aid in reducing overall antimicrobial resistance [11,17]. Pertinently, there is a need for improvement in training and education in laparoscopic surgical techniques, especially for adrenal surgery in developing countries [20]. This will help to improve outcomes, reduce complications, and decrease the length of hospital stay [11].

A retrospective and cross-sectional study design accounts for one of the limitations of our study. Therefore, associations and predictors of the complications related to open adrenalectomy could not be established. Moreover, data from multiple public or private centers could have expanded the results of our study. Nonetheless, our study provides important conclusions for the betterment of general surgery, especially in developing countries where laparoscopic techniques are less frequently used. Further research and quality improvement projects are required to decrease the incidence of these complications and improve overall outcomes.

## Conclusions

Surgical site infections, pulmonary infections, retroperitoneal hematoma, deep venous thrombosis, and sepsis were common complications of open adrenalectomy observed in a public sector hospital in a developing country. These complications lead to morbidity and abysmal outcomes. The high prevalence of these complications necessitates the need for improvement in patient optimization, meticulous intraoperative hemostasis management, the development of strict infection control measures, and antibiotic surveillance. Moreover, a multidisciplinary approach is of paramount importance for optimal postoperative management and better outcomes.
